# High-density EEG signature of NREM sleep parasomnia episodes

**DOI:** 10.1038/s41598-026-41601-4

**Published:** 2026-04-27

**Authors:** A. Valomon, I. De Cuntis, K. Nakamura, B. A. Riedner, S. Jones, M. Bazalakova, D. T. Plante, G. Tononi, M. Boly

**Affiliations:** 1 Psychiatry-Wisconsin Institute for Sleep and Consciousness, University of Wisconsin-Madison, Madison, WI 53719 USA; 2 School of Medicine and Public Health, University of Wisconsin-Madison, Madison, WI USA; 3 Neurology, University of Wisconsin-Madison, Madison, WI USA

**Keywords:** Neurology, Neuroscience, Physiology

## Abstract

NREM (non-rapid eye movement sleep) Parasomnia Episodes (PE) such as confusional arousals and sleep-talking represent abnormal wake-like behaviors initiated during sleep, with a known admixture of localized wake-like and sleep-like activities. Using high-density EEG (hdEEG), we quantified whole-scalp topographical EEG differences during PE compared to wakefulness and sleep. Twenty-two adults with NREM sleep parasomnia underwent 256-electrode hdEEG over two nights: a baseline night with ad-libitum sleep and an intervention night with recovery sleep after 25 h sleep deprivation. 79 PE occurred spontaneously or when triggered from NREM sleep during intervention nights. Spectral power of PE, sleep, and wake EEG was compared. Data from 70 PE (in 20 subjects) were of sufficient quality to be compared to sleep (matched for consciousness and sleep stage when possible) and wake. Delta and theta power during PE were significantly higher than wake but significantly reduced compared to sleep in central scalp regions. Additionally, beta/delta ratio values during PE were globally higher than during wake, but globally lower than during sleep. These findings suggest NREM PE feature a unique pattern of mixed cortical arousal and sleep, reflecting abnormal local regulation of sleep–wake boundaries in arousal disorder patients.

**Supplementary Information:**

The online version contains supplementary material available at 10.1038/s41598-026-41601-4.

## Introduction

NREM parasomnias are characterized by the concurrent presence of distinct states of wakefulness and sleep. Clinically, these conditions are often classified as either arousal disorders or as difficulties in the transition between sleep and wakefulness. Such disturbances lead to incomplete awakenings during sleep^1^. These parasomnias typically occur during N2 and N3 sleep stages and encompass a range of behaviors, including sleepwalking, sleep-talking, confusion upon awakening, night terrors, and more atypical variants such as sexsomnia and sleep-eating^2–4^.

Although NREM parasomnias are more prevalent in children, they are more common in adults than is generally acknowledged. While parasomnias in children are typically benign, those observed in adults can pose substantial risks, leading to unintentional engagement in hazardous activities, self-injury, harm to others, or property damage during episodes^6^. The disruption of sleep caused by these events, along with an increased frequency of both behavioral and non-behavioral arousals, contributes to cognitive deficits and diminished daytime alertness in individuals with NREM parasomnias^7–11^. While numerous factors—behavioral, genetic, pharmacological, and environmental—contribute to the development and manifestation of these disorders^5^, their underlying neural mechanisms remain insufficiently understood.

A central issue in the study of NREM parasomnias is the question of whether alterations in slow waves during sleep play a direct causal role in these disorders or whether they simply reflect a secondary consequence of abnormal brain processes^12^. Understanding the relationship between brain activity and the behaviors exhibited during parasomnia episodes remains a significant challenge. Previous studies employing intracranial EEG and SPECT imaging have documented dissociative states during parasomnia events, with persistent sleep patterns in fronto-parietal regions of the brain, while sensorimotor areas paradoxically show activation^13–16^. More recent research using broad-band spectral analysis with low-density EEG (19 channels) in a larger cohort of individuals with disorders of arousal (DoAs)^17^ has largely focused on brain activity preceding parasomnia episodes, rather than on the distinct spectral signatures inherent to the episodes themselves. Electroencephalographic studies aggregating data from various forms of NREM DoAs have identified abnormalities in EEG power within the slow-wave frequency range (delta, 1–4 Hz), characterized by abnormal overnight accumulation and an increased presence of hypersynchronous delta waves^18–23^. Moreover, parasomnia patients exhibit a notable propensity for local activation of motor and limbic thalamocortical circuits during sleep, coupled with lower arousal thresholds^24^.

A recent study^23^ explored the neural correlates of parasomnia episodes (PE) using high-density electroencephalography (hdEEG) in combination with a sleep deprivation–serial awakening approach. This investigation primarily observed spontaneous parasomnia events occurring predominantly during N3 sleep in a group of 20 individuals with DoAs, excluding those with sleep talking episodes. Analysis of delta band power distribution across various brain regions revealed consistently reduced delta power during parasomnia episodes when compared to normal sleep, particularly in the fronto-polar regions. Additionally, delta power was consistently elevated across sensory and motor cortices during parasomnia episodes relative to wakefulness.

The present study expands upon these findings by employing high-density EEG with a topographical resolution provided by 256 electrodes in a sample of 27 individuals exhibiting a plurality of DoAs, the majority of whom present with sleep-talking episodes. In addition, we collected reports of PE recall immediately following awakening,  as done in previous studies ^7,25–27^, and compare them to high-density EEG spectral findings.

## Results

### Demographics of study participants

The recruited parasomnia subjects comprised young adult males and females with a history of sleepwalking or sleep-talking, who reported a high frequency of parasomnia episodes, as assessed by the Paris Arousal Disorder Severity Scale (PADSS) scale and self-reported questionnaire items grouped in the standardized Wisconsin Sleep History Questionnaire. These subjects also exhibited more symptoms of insomnia, restless legs syndrome (RLS), and narcolepsy compared to matched controls (see Table 1).

**Table 1:** Demographics of study participants. Controls and participants were matched for age, gender and BMI. They were mostly right-handed, with no CNS (Central Nervous System) medication except in 2 cases (venlafaxine - lorazepam) and with few comorbid psychiatric disorders (5 anxiety, 2 depression and 1 ADHD). Mean ± STD are presented. One-way ANOVA for effect of group. For categorical values, Chi-square tests were used. Details of tests below. Parasomnia screening was done with the PADSS (Paris Arousal Disorders Severity Scale). Health-related quality of life was obtained with the SF36 questionnaire (36-Item Short Form Health Survey) (Total score presented in table). Depression was assessed with the IDS (Inventory of Depressive Symptoms) and anxiety using a modified version of the PSWQ (Penn State Worry Questionnaire). Sleep inertia from the SIQ (Sleep Inertia Questionnaire). Sleepiness and its daytime effects and fatigue were assessed with the ESS (Epworth sleepiness scale), the FOSQ (Functional Outcomes of Sleep Questionnaire) and the FSS (Fatigue Severity Scale) respectively. Symptoms of sleep disorders were described and grouped to assess the likelihood and severity of 5 major sleep disorders (insomnia, sleep apnea, parasomnias, narcolepsy, and restless leg syndrome (RLS)). The insomnia scale corresponded to the ISI (Insomnia Severity Index scale). Others were custom-made at the Wisconsin Sleep Clinics, with items rated from 1 to 5, with 8 items for apnea, 12 for parasomnias and 15 for narcolepsy. RLS screening consisted of 3 yes/no questions, and "some symptoms of RLS" were defined from one yes answer. Sleep quality, duration and circadian preference were assessed using the PSQI (Pittsburgh Sleep Quality Index), the reported sleep duration and the calculated sex and age corrected chronotype index from the MCTQ (Munich ChronoType Questionnaire).

	Controls 12	Parasomnia 22	F (or Chi- sq)	*p*-value
Gender ratio (women/men)	5/7	9/13	0.0018	0.96
BMI	23.42±3.81	24.37 ± 3.39	0.63	0.43
Age	26.25±3.77	27.31 ± 11.30	0.75	0.39
Comorbid psychiatric diseases (yes/no)	1/11	5/17	1.11	0.29
CNS medication (yes/no)	0/12	2/20	na	
Handedness (L/R)	1/11	1/21	0.20	0.65
Parasomnia score (PADSS)				
A-Behaviors	0.0 ± 0.0	4.5 ± 3.5	20.08	**< 0.001**
B-Frequency	0.0 ± 0.0	3.0 ± 1.3	82.70	**< 0.001**
C-Effects	0.0 ± 0.0	2.4 ± 1.3	40.61	**< 0.001**
Self-reported health (SF36)	84.8 ± 8.6	80.2 ± 11.8	1.41	0.24
Depression (IDS)	7.4 ± 3.6	10.2 ± 6.0	2.10	0.16
Worry	35.3 ± 9.8	39.0 ± 9.8	1.11	0.30
Sleep inertia (min)	11.3 ± 10.5	20.6 ± 19.4	2.34	0.14
Functional sleep outcome score (FOSQ)	42.8 ± 9.4	50.3 ± 13.0	3.02	0.09
Sleepiness (ESS)	7.0 ± 3.1	7.8 ± 3.3	0.45	0.51
Fatigue (FSS)	23.3 ± 9.9	24.1 ± 10.9	0.04	0.83
Self-reported symptoms				
Insomnia	3.7 ± 3.3	7.4 ± 4.9	5.41	**0**.**03**
Sleep apnea	14.2 ± 5.1	13.5 ± 3.2	0.22	0.64
Parasomnia	15.5 ± 2.8	26.7 ± 6.8	28.99	**< 0.001**
Narcolepsy	15.1 ± 1.8	19.5 ± 5.9	6.19	**0**.**02**
Restless legs (none/some)	11/1	13/9	3.97	**0**.**05**
Sleep quality (PSQI)	4.3 2.5	4.6 2.6	0.10	0.75
Average sleep duration (MCTQ)	8.3 ± 1.1	8.3 ± 4.0	0.00	0.98
Chronotype (MCTQ)	3.9 1.1	4.4 2.3	0.81	0.38

For analysis purposes, the parasomnia subjects were pooled together, though they could be further categorized into “sleep-talkers only” (*n* = 3; subjects who exclusively reported sleep-talking episodes) and those with DoA, further subdivided into past/rare (*n* = 6) and current/frequent (*n* = 13) episodes. All past DoA subjects reported at least current sleep-talking episodes. In addition to confusional arousals and sleepwalking, some DoA subjects also reported past or present episodes of sexsomnia (*n* = 3) and night terrors (*n* = 7), but no episodes of sleep-eating. A family history of parasomnias was reported by 10 out of 22 subjects (see Table 2). Most subjects experienced parasomnia episodes since childhood or adolescence (*n* = 20), with identified triggers including stress (14), sleeping in new environments (12), sleep deprivation (12), alcohol (4), and strong emotions (3). The frequency of episodes reportedly increased during college or graduate school for some subjects (*n* = 10). Additional triggers for sexsomnia included sleeping with a new partner, skin touch, or heavy exposure to pornography. Supplemental Fig. 1 highlights several other variables, such as mental and physical health, depression, worry, fatigue, and sleep inertia, which were found to be worse in DoA subjects compared to past DoA subjects, sleep-talkers, and controls.

**Table 2:** Details on parasomnia episodes with or without conscious experience. The table presents each parasomnia organized per subject, origin of the NREM PE (parasomnia episodes) occurred within the experimental setting (baseline or recovery sleep, BS, RS); sleep stage of the PE (N2 or N3); unique parasomnia code, comments on PE recording quality or matching possibilities—states whether the PE was unusable for analysis (*n* = 9) and whether it could not be matched with a sleep period (*n*=8); presence of consciousness report (divided into CE: Conscious Experience and CNR: Conscious Experience with No Recall. N represents parasomnia with no conscious recall upon awakening); subtype of disorder presented by the participant (disorder of arousal—DoA, past DoA or sleep talking—ST); presence of a familiar history of parasomnia; type of recorded PE (sleep-talk—ST, confusional arousal (CA): simple or complex); rating on the amount of sounds and movement occurred during PE. Speech and body movements were separately rated on a scale from 0 to 2 for speech and 0 to 3 for movements. Speech scores consisted of: 0 for silent episode, 1 for episodes consisted of short sounds (like “Hmm”, “Wow”) or words (like “no”, “three”), and 2 corresponded to longer outputs forming part or complete sentences (completely or partially intelligible or gibberish whispering). The movement scale started at 0: for sleep-talk episodes that had no body movements except face and lip muscles movements; 1 eyes are open; 2 eyes are open with clear head movement such as looking around, small hand movements; 3 purposeful movements, larger body movements. Simple CA had movement ratings from 1 to 2 and complex CA of 3, with speech scores from 0 to 3. Sleep-talk episodes had movement rating of 0 and speech rating of 1 to 2.

Subject	Night	Sleep stage	Unique parasomnia code	Comments on parasomnia	Conscious ness report	Subtype	Familiar history of parasomnia	Type	Speech rating	Movement rating
P001	RS	N2	60	Unusable	N	Past DoA	Yes	Simple CA	1	1
P002	RS RS RS	N3 N3 N3	86 88 90		. N N	DoA	No	ST Simple CA Complex CA	1 0 0	0 1 3
P003	BS RS	N3 N3	128 135		. N	Past DoA	No	ST Simple CA	2 1	0 2
P004	RS RS RS RS	N3 N2 N3 N2	34 32 33 28	Unusable Unusable	N . N CE	ST	Yes	Simple CA Complex CA Complex CA Simple CA	1 2 2 1	2 3 3 2
P005	RS RS RS RS RS RS RS	N3 N2 N3 N2 N3 N2 N3	46 51 43 52 41 45 49	Unusable	N CE CNR CE CE CE CE	DoA	No	Simple CA Complex CA Complex CA Complex CA Complex CA ST Complex CA	0 1 2 0 2 2 1	2 3 3 3 3 0 3
P006	RS BS RS BS RS RS	N3 N3 N3 N3 N3 N3	77 68 74 69 75 78		CE . CNR . CE CE	DoA	Yes	Complex CA Simple CA Simple CA Simple CA Complex CA Complex CA	0 0 0 1 2 2	3 1 2 2 3 3
P007	RS RS RS	N2 N2 N3	114 111 113		CE . CE	DoA	Yes	ST Simple CA ST	1 1 2	0 2 0
P008	RS RS RS	N2 N3 N2	160 156 159	Unusable + 5 sleep-talk PE from REM	CE N N	DoA	No	ST ST Complex CA	2 2 2	0 0 3
P009	RS RS RS RS	N3 N2 N3 N3	10 5 4 8		CE N CNR CNR	Past DoA	No	Simple CA Simple CA Simple CA ST	1 1 1 2	1 1 1 0
P010	RS RS	N3 N3	124 126		N N	DoA	Yes	Simple CA Simple CA	0 0	2 2
P011	BS BS RS RS RS RS	N3 N3 N3 N3 N3 N3	11 12 17 19 15 18	Unusable Unusable	. . N N N N	Past DoA	Yes	Simple CA Complex CA Simple CA Simple CA Complex CA Complex CA	0 2 1 1 2 1	2 3 2 2 3 3
P012	RS RS RS RS	N3 N3 N3 N2	143 140 148 147		N N N N	Past DoA	No	Complex CA ST Simple CA Simple CA	2 2 1 0	3 0 1 1
P013	RS RS RS RS BS BS	N3 N2 N3 N3 N3 N2	182 184 178 180 176 177	no match no match	CE CE N N . CE	DoA	No	Complex CA ST ST Complex CA Complex CA Complex CA	2 2 2 2 2 2	3 0 0 3 3 3
P014	BS RS RS	N3 N2 N3	186 190 189		CE CE CE	DoA	No	ST ST Complex CA	1 1 2	0 0 3
P015	BS BS BS BS BS RS RS	N3 N2 N3 N2 N2 N3 N3	252 254 255 257 256 271 274		CE N CE N CE N N	DoA	Yes	Simple CA ST ST ST ST ST ST	0 1 2 1 2 1 1	1 0 0 0 0 0 0
P016	BS BS	N2 N3	291 292	No match No match	N N	DoA	No	ST Complex CA	2 2	0 3
P017	BS	N3	338	Unusable	N	DoA	No	Simple CA	0	1
P018	BS BS RS RS RS RS BS	N2 N2 N3 N3 N3 N3 N2	348 351 354 356 359 360 352	Unusable	N CE N N CE N N	DoA	Yes	ST Complex CA Simple CA Complex CA Complex CA ST ST	1 2 0 1 2 2 1	0 3 2 3 3 0 0
P019	RS RS RS BS BS BS	N3 N2 N2 N2 N2 N2	374 375 378 366 367 368	Unusable no match no match no match	CE CE CE CE CE CE	Past DoA	No	Complex CA ST ST ST Simple CA ST	1 1 1 1 1 1	3 0 0 0 1 0
P020	RS BS	N2 N3	385 379	no match	CE CE	DoA	Yes	ST Simple CA	1 1	0 2

### Sleep characteristics

Controls and parasomnia subjects exhibited similar sleep architecture and composition (see Table 3 for “group” p-values). The only significant effect of the “group” variable was found in the arousal index (*p* = 0.05), with more arousals observed in controls, likely due to their higher tendency to present with sleep apnea (chi-square *p* = 0.06). However, parasomnia subjects showed an abnormally high number of arousals from N3 sleep, as noted by the reviewing physician (chi-square *p* = 0.01), consistent with their parasomnia diagnosis.

**Table 3:** PSG characterization of study participants. Means ± STD are presented. One-way ANOVA was applied to calculate effect of group in baseline night for sleep disorder screening aspects (only assessed during baseline night). Chi-square tests were used for categorical measures such as RSWA, Apnea diagnosis and N3 arousals frequency. Repeated-measure ANOVA were performed for effect of group and night on other measures. Note: Baseline specific PSG (polysomnography) assessments were not obtained in all subjects (see details in in Material and methods). Therefore, in the lower part of the table, means and numbers correspond to 12 controls and 16 (instead of 22 subjects in the parasomnia category.) Percentage time in N1, N2, N3, REM and wake (WASO) stages are indicated. TST: total sleep time (in min). Sleep efficiency was calculated as TST divided by time in bed. Sleep latency was defined as latency to reach stage N2, SWS latency to N3, and REM latency to REM stage. Arousals (defined as interruption of sleep lasting 3 to 15 s) in total sleep, REM or NREM sleep are indicated as number of arousals per hour of sleep. Sleep quality measures, only assessed during the baseline sleep, include: apnea-hypopnea index (AHI), respiratory disturbance index (RDI), periodic leg movement index (PLMI) per hour of sleep, mean heart rate during TST (HR). Subjects were further divided into presenting or not with REM sleep without atonia (RSWA), with mild to moderate apnea (Apnea) or with abnormally frequent N3 arousals as determined by the physician (N3 arousals).

	Baseline night		Recovery sleep								
	Control	Parasomnia	Control			Parasomnia			*p* values		
	12	22	12			22			Group	Night	Interaction
N1 (%)	6.0% ± 2%	4.2% ± 2%	6.2% ± 4%			5.8% ± 3%			0.18	0.23	0.14
N2 (%)	46.1% ± 8%	45.3% ± 7%	45.8% ± 9%			40.7% ± 10%			0.30	0.10	0.50
N3 (%)	18.1% ± 6%	18.8% ± 6%	24.4% ± 7%			19.9% ± 6%			0.31	**0**.**03**	0.18
REM (%)	17.3% ± 6%	21.3% ± 5%	12.2% ± 5%			14.5% ± 7%			0.12	**< 0.001**	0.55
WASO (%)	12.6% ± 9%	10.5% ± 7%	11.3% ± 6%			19.1% ± 14%			0.37	**0**.**03**	0.17
TST (min)	459 ± 65	470 ± 58	329 ± 27			373 ± 57			0.07	**< 0.001**	0.48
Sleep efficiency (%)	85.7% ± 9%	85.6% ± 8%	85.1% ± 5%			79.7% ± 14%			0.43	0.14	0.30
Sleep onset to N2 (min)	15.4 ± 9.2	15.1 ± 15.4	5.0 ± 4.8			4.2 ± 2.9			0.83	**< 0.001**	0.93
SWS latency (min)	32.1 ± 11.5	34.9 ± 40.4	14.7 ± 6.3			12.3 ± 5.5			0.98	**< 0.01**	0.65
REM latency (min)	138.7 ± 48.5	128.3 ± 58.3	101.1 ± 43.0			65.6 ± 29.2			0.07	**< 0.001**	0.26
Arousal index	9.76 ± 5.2	7.30 ± 4.2	5.92 ± 3.7			3.33 ± 1.8			**0**.**05**	**< 0.001**	0.32
NREM arousal index	10.47 ± 6.7	6.53 ± 5.8	4.12 ± 4.0			2.80 ± 1.5			0.12	**< 0.001**	0.68
REM arousal index	12.11 ± 8.1	10.51 ± 7.1	8.62 ± 10.7			6.98 ± 7.6			0.65	**< 0.01**	0.50
AHI (/h)	5.2 ± 7.4	4.4 ± 5.8							0.86	0.36	
RDI (/h)	11.4 ± 9.3	8.1 ± 7.7							3.53	0.07	
PLMSI (/h)	2.0 ± 4.2	1.2 ± 2.8							1.01	0.32	
HR (bpm)	54.1 ± 7.0	60.3 ± 8.8							2.43	0.13	
RSWA (none/noted)	11/1	10/4							1.70	0.19	
Apnea (none/mild to moderate)	8/4	5/11							3.46	0.06	
N3 arousals (normal/high)	11/1	7/8							6.07	**0**.**01**	
PLMD/RLS diagnosis	0	0									

On average, 8 h of sleep were recorded for the baseline night, compared to nearly 6 h for the recovery night. Following 25 h of wakefulness, sleep initiation was faster during the recovery night, with shorter sleep latency to N2, SWS, and REM sleep. The relative time spent in N3 sleep increased, while the time spent in REM sleep decreased. Wake After Sleep Onset (WASO) was artificially elevated due to repeated awakenings and interrogations, whereas arousals decreased (number per hour of Total Sleep Time (TST), per NREM sleep, or per REM sleep), indicating less fragmented sleep. None of these homeostatic changes significantly differed between controls and parasomnia subjects (“group” × “night” interaction *p* > 0.05; see Table 2).

### Parasomnia episodes

#### Characteristics of parasomnia episodes captured with hdEEG

A total of 79 NREM parasomnia episodes were obtained from two experimental nights involving 20 patients (see Supplemental Table 1). This resulted in an overall success rate of 91% for triggering parasomnias in susceptible subjects. Four patients experienced fewer than one parasomnia episode (PE) over these two nights, while seven patients had between six and seven PEs. A total of 23 PEs were recorded during baseline (BS) nights, and 56 were recorded during sleep-deprived (SD) nights. Of these episodes, 39 were spontaneously triggered, 34 were induced by experimental auditory stimuli, and 6 were caused by environmental noises, such as high heels and flushing sounds.

**Fig. 1:**
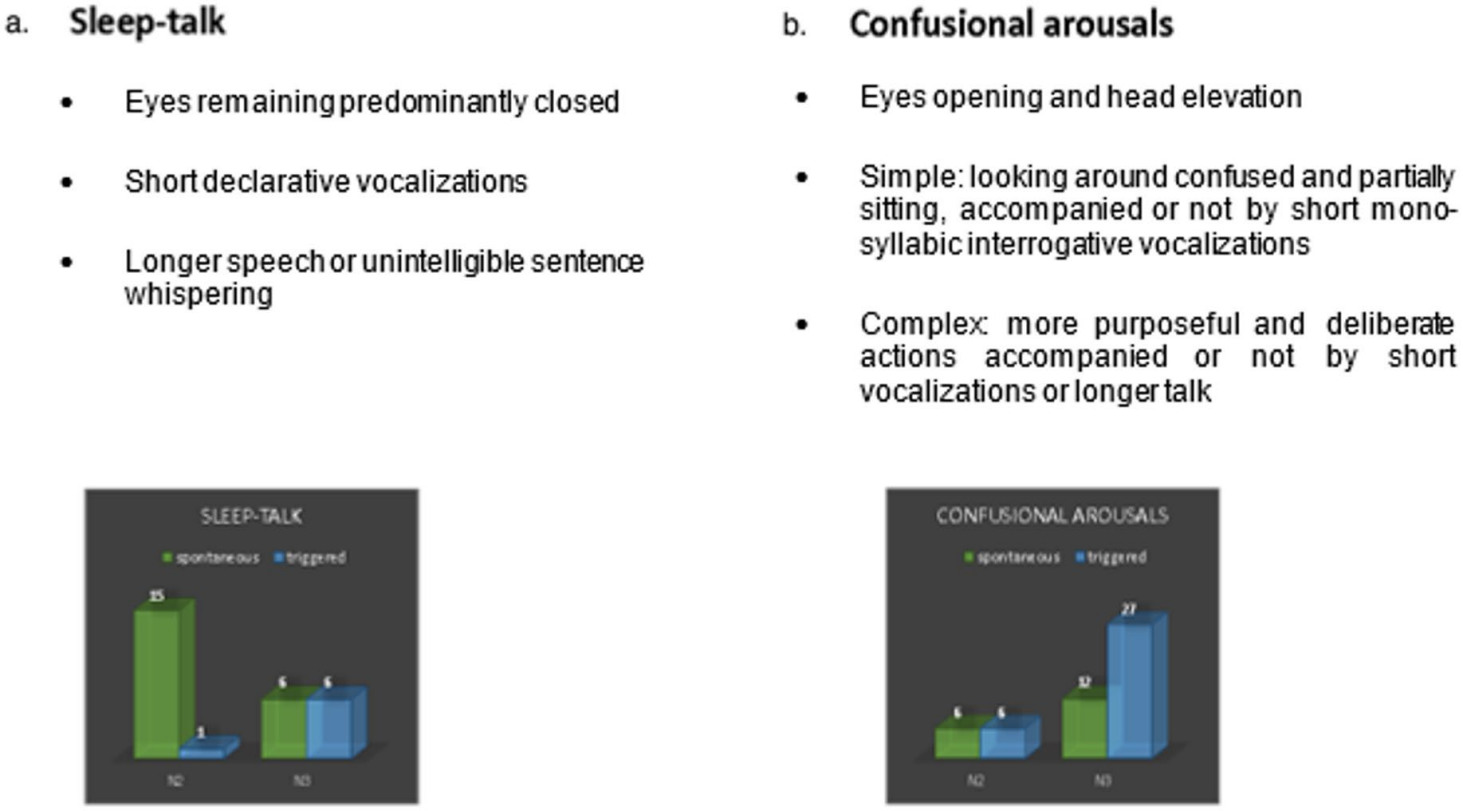
Characteristics of recorded Parasomnia Episodes. 79 episodes could be further distinguished as “sleep-talk” (28) or “confusional arousals” (54). **a**. Represents features of sleep-talking episodes (ST), with blue color in the bar chart indicating episodes triggered by sounds, while green indicating spontaneous ones. Sleep stages the episode originated from are indicated (N2 o N3). **b.** Shows confusional arousals (CA) features. Blue and green, as well as sleep stages presented in pie charts follows the logic of a.

Out of the total PEs, 28 were sleep-talking episodes, which involved minimal body movement. In only 7 instances the patients’ eyes were open, and 15 episodes consisted of short, single-word expressions, while 13 involved comprehensible or unintelligible sentences. These episodes generally occurred during N2 sleep (61%) and were seldom triggered by sound (25%) (see Table 3). Upon awakening, an experience was reported in most cases (56%) (see left side of Fig. 1).

Confusional arousals were categorized into simple (28 instances) and complex (26 instances). These ranged from simply looking around with eyes open and appearing confused, to partially sitting up or lifting the neck, with or without brief monosyllabic vocalizations. More complex episodes involved purposeful and deliberate actions with eyes open, accompanied by short or long sentences. The majority of these arousals originated from N3 sleep (84%) and were triggered by auditory stimuli (65%). An experience was reported in 47% of the cases, with a notable difference between simple CAs (33%) and complex CAs (61%) (see right side of Fig. 1).

In all types of parasomnia episodes, consciousness during the episode was more frequently retained when the parasomnia emerged from N2 sleep (Fig. 1). Detailed information on these parasomnia episodes (PEs) with conscious report is available in Supplemental Tables 2 and summarized here.

Out of 36 of such episodes, participants knew they were having an experience, but they could not describe it in 4 cases. On the other extreme, 10 had complex experience and described it in detail. Interestingly, 11 episodes were related to the sleep lab environment and study. In 20 episodes the experience resembled something the participant had previously encountered. Notably, in 15 cases, participants reported being aware of their behavior during the episode. Emotions during the parasomnias were generally neutral, with only three instances reporting negative or very negative emotions.

#### Global spectral signature of parasomnia episodes

**Fig. 2:**
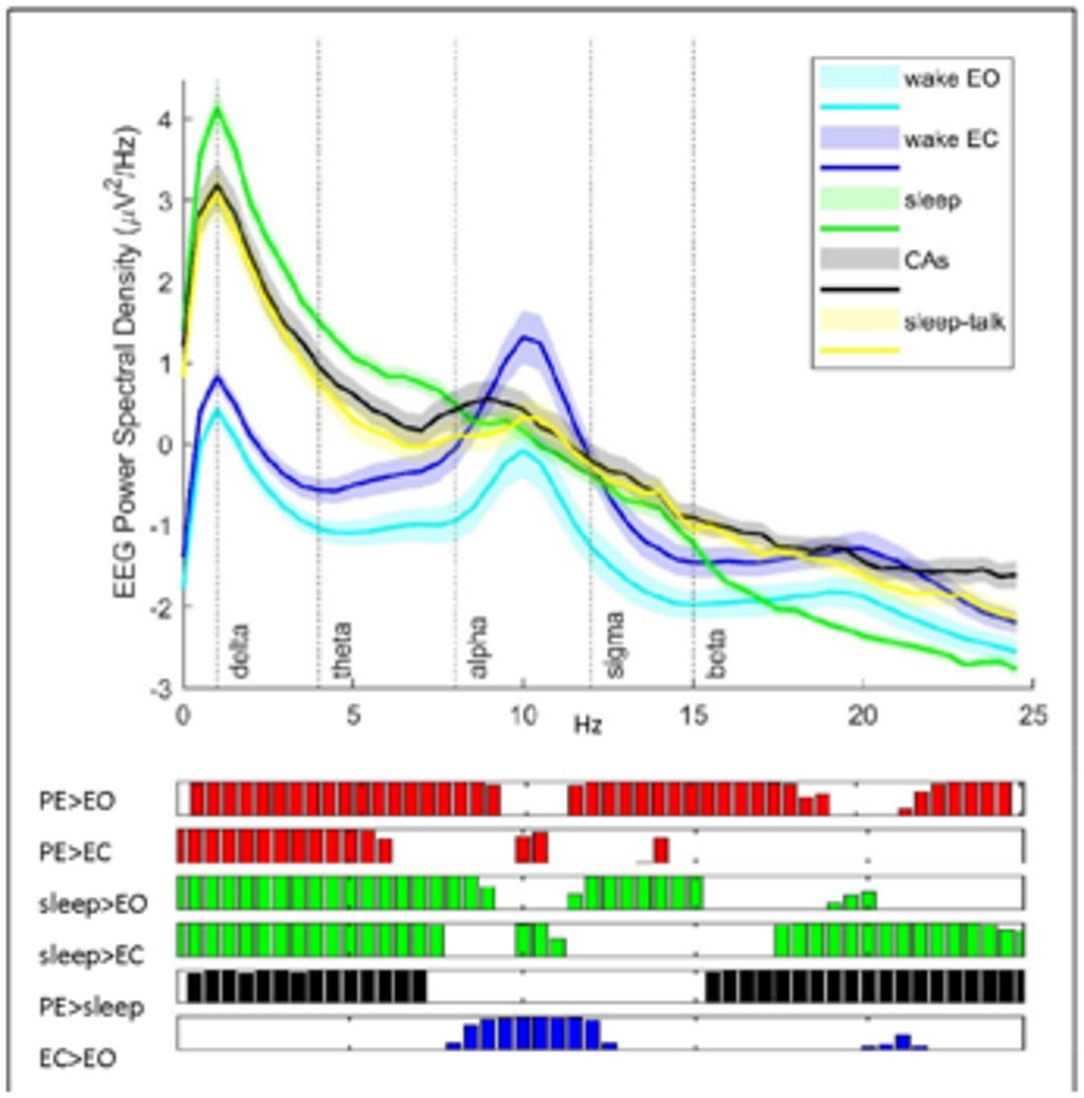
Mean scalp power of the different vigilance states. PE (parasomnia episodes) and ST (sleep talking) episodes were split in the graph but grouped for analyses. Tukey-Kramer post-hoc *p*<0.05 are displayed as bar charts for specific comparisons, below the graph: for instance, ‘PE> EO’ represents the post-hoc values for the comparison of grouped PEs vs. wake Eyes Open (EO). PE were matched per subject to wake EEG recorded at 18 h of wakefulness and to sleep EEG. EC: wake Eyes Closed.

The mean scalp power from 0.5 to 25 Hz for sleep, wakefulness, and parasomnia episodes is displayed in Fig. 2. As anticipated, NREM sleep exhibited higher delta and theta power compared to wakefulness, as well as lower beta power. A peak in the sigma range during NREM sleep reflected the presence of sleep spindles. Additionally, recordings during wakefulness with eyes open differed from those with eyes closed, showing a decrease in alpha power. Parasomnia episodes exhibited diminished delta and theta power compared to sleep, yet these levels were significantly greater than those observed during wakefulness. Interestingly, parasomnias had similar power to sleep in the alpha and sigma power ranges. However, they deviated from sleep in terms of higher frequencies, with beta power comparable to that seen in wakefulness.

In summary, parasomnias exhibited similarities with sleep at the whole scalp level for low-frequency activity, while mirroring wakefulness for high-frequency activity.

#### Topographical maps of parasomnia episodes

**Fig. 3:**
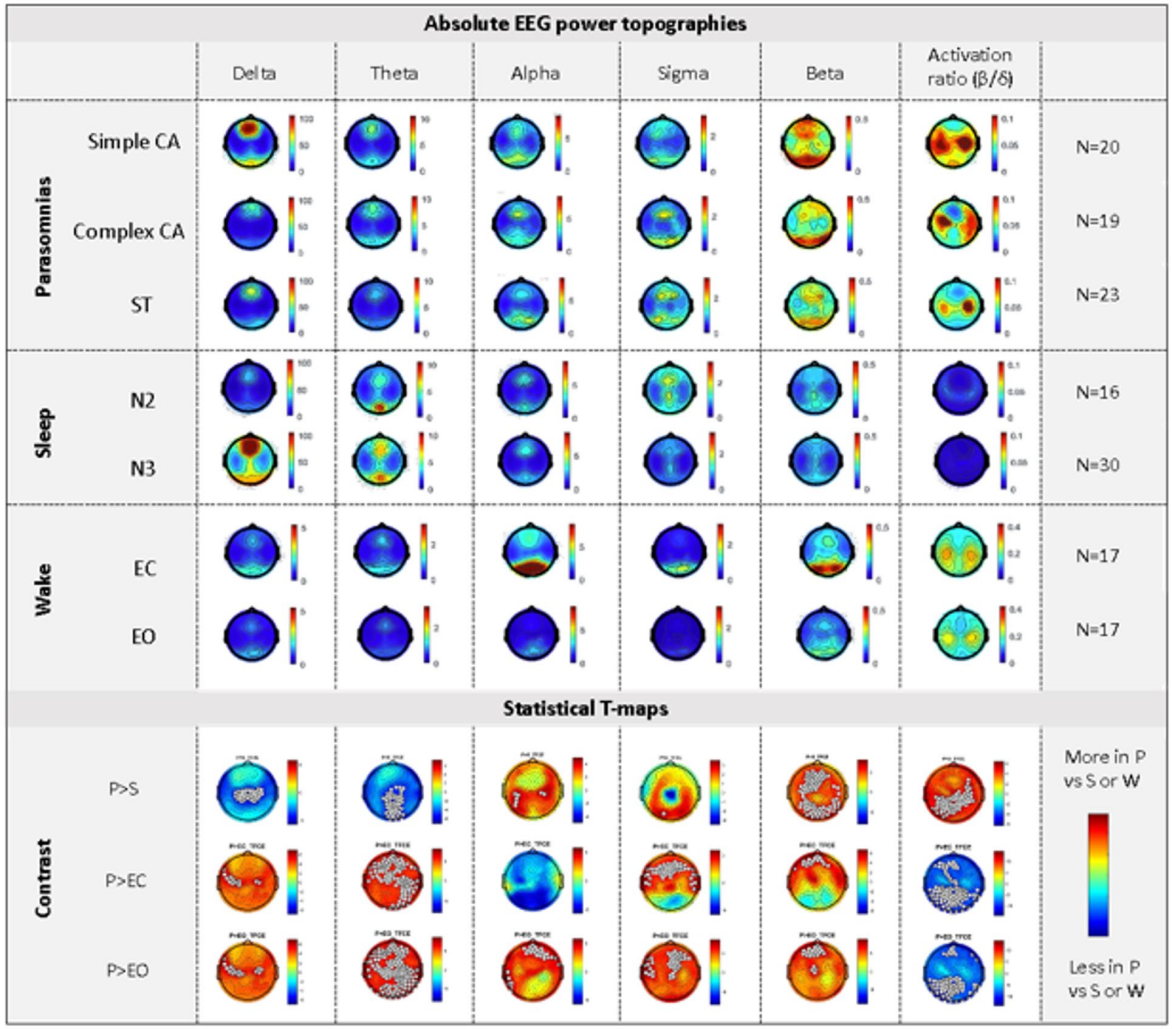
Topographical characteristics of parasomnia compared to sleep and wake. Top. Absolute topographical power densities of EEG frequency bands [delta (1–4 Hz), theta (4–8 Hz), alpha (8–12 Hz), spindle (12–15 Hz), beta (15–25 Hz), beta/delta ratio (a marker of cortical arousal)], are displayed, split by vigilance states. Parasomnia were split by types (complex confusional arousal (Complex CA), Simple CA, sleep talking (ST)), sleep by stage (N2/N3), and wake by condition (wake eyes open (EO)/wake eyes closed(EC)). Counts of analyzed data are indicated on the right. Bottom. Spectral powers of EEG signal were compared between states using paired t-tests. All results were thresholded at *p*<0.05 corrected for multiple comparison using threshold-free cluster-enhancement method (TFCE). White dots indicate *p* < 0.05. Color coded scale on the right indicates direction of contrast.

Using high-density EEG topographical analyses, we examined the local topographical distinctiveness of electroencephalographic patterns during parasomnias in comparison to sleep and wakefulness (Fig. 3). Compared to sleep, PE were characterized by decreased slow-wave activity (SWA) localized to medial centro-occipital regions (p < 0.05, TFCE corrected). This central cluster overlaps with the medio-parietal ‘hot spot’ highlighted by Castelnovo^20^, identified as less deactivated during baseline sleep in parasomnia patients compared to controls. In contrast, frontal SWA was similar between PE and sleep. The collected data exhibited a similar pattern within the theta range as well, showing significant medio-occipital theta power reduction in PE compared to sleep (*p* < 0.05, TFCE corrected), with the effect being more spatially extended across the cortex than in the delta range. Furthermore, as in the delta range results, the frontal cortex activity was similar between PE and sleep for theta activity.

Coherently, with respect to the beta range, fronto-parieto-occipital cortices exhibited increased activity (beta power) during parasomnia episodes relative to sleep. Overall, posterior cortical regions were relatively more activated during parasomnia episodes compared to sleep (*p* < 0.05, TFCE corrected), while frontal activity displayed a mixed pattern—resembling sleep for low frequencies, while exhibiting wake-like signatures in higher frequencies.

The comparison between parasomnias and wakefulness revealed significant results as well. Higher delta activity was found during parasomnias compared to wakefulness (both with eyes closed and open), especially significant over left centro-frontal derivations (*p* < 0.05, TFCE corrected). Between-state differences were particularly widespread within the theta range, with PE theta activity being higher than wakefulness across the scalp (*p* < 0.05, TFCE corrected).

Power during PE was also higher than during wakefulness in the sigma range, particularly across medio-frontal regions (*p* < 0.05, TFCE corrected), suggesting a higher occurrence of sleep spindles during parasomnias than during the wake state. Finally, PE exhibited significantly higher frontal beta activity compared to wakefulness (*p* < 0.05, TFCE corrected). Despite the use of state-of-the-art data-cleaning techniques, residual muscular artifacts may still have affected the beta range.

Finally, comparison of absolute power in the delta frequency range highlighted interesting differences in the electroencephalographic signatures of different parasomnia types. Specifically, comparing sleep-talking episodes with simple and complex CAs revealed that the simpler the episode, the higher the delta power. Functionally, less complicated parasomnias were associated with lower frontal activity. Simple CAs presented the highest amount of SWA. However, due to the small sample size, these comparisons did not reach statistical significance.

## Discussion

We employed state-of-the-art high-density EEG and independent component analysis to explore the spectral characteristics of parasomnia episodes compared to sleep and wake states in a cohort of 22 individuals with parasomnias, encompassing a range of episode types, such as confusional arousals and sleep-talking. Our findings show that delta and theta power during PE were significantly higher in central regions when compared to the wake state, while delta and theta power was lower in central regions and occipital alpha power was higher compared to sleep. The primary conclusion of this study validates the dualistic nature of parasomnias, positioning them as phenomena that lie in an intermediate physiological state between sleep and wakefulness.

By using the advanced high spatial resolution of 256-electrode high-density EEG recordings, combined with independent component analysis, we were able to examine EEG power during artifact-free segments, despite occasional movements during some recorded epochs. Unlike previous studies, our protocol incorporated serial awakenings coupled with sleep deprivation to maximize the occurrence of parasomnias while maintaining the global sleep structure during recovery nights, through an adequate distance from baseline-night laboratory sessions.

The decrease in slow-wave activity (SWA) and theta power in the centro-parietal region during PE, relative to sleep, might be related to the motor-limbic behavioral manifestations observed during PE.

As anticipated, this region appears to be in a wake-like state during the episodes. The overlap observed between the strongest effect cluster in our results and the centro-parietal ‘hot zone’ identified by Castelnovo^20^—which is less deactivated in normal sleep of patients compared to controls—suggests that localized reductions in delta power in these regions, even during baseline sleep, could serve as a potential biomarker for parasomnia risk. Future studies involving larger cohorts could examine the correlation between SWA reduction in these regions and the intensity or severity of parasomnia episodes, which could provide significant insights.

A key aspect of our findings is the novel observation that no significant differences in frontal SWA (indicative of sleep intensity) were found between parasomnias and sleep within the same patients. This supports the dissociative nature of parasomnias, where motor-limbic activation occurs simultaneously with frontal inhibition, leading to a dormant signature.

### Motor activation

The activation of motor, premotor, and supplementary motor areas during PE strongly converges with the frequent movements exhibited by our patients during parasomnia episodes. Functionally, activation of the posterior parietal region could also account for specific somnambulistic skills, such as obstacle avoidance. This is supported by recent findings highlighting the contribution of posterior parietal regions to navigation abilities in feline locomotion models^34,35^.

A detailed comparison of pre-SMA activity over time during parasomnias versus other sleep periods, along with causal connectivity analyses between pre-SMA and motor cortex activity, would clarify the role of volition in initiating motor actions during parasomnias. Differences in occipito-parietal power shifts might vary between periods like falling asleep—where volition decreases despite the presence of consciousness—and parasomnias, which could feature high motor volition and low consciousness. These investigations, combined with new insights into the neural correlates of parasomnias, could clarify the contentious issue of pre-SMA activity signaling motor intentions before conscious awareness. This would shed light on the role of volition in dramatic changes to motor plans, an important aspect of the precedence of knowledge over will, as discussed by Mainieri et al.^17^.

Importantly, including sleep-talking episodes in the EEG analysis—an approach not previously utilized in NREM sleep parasomnia literature—may help explain the reduction in parieto-temporal SWA observed during parasomnias in our cohort. This reduction could be partially attributed to activity in the temporal and angular gyrus and parietal lobule, regions with established roles in language processing^36,37^.

### Limbic network activation

We observed medio-parietal activation and prefrontal deactivation during parasomnia episodes, compared to sleep. The medial parietal activation, alongside prior reports of cingulate, insular, and temporo-parietal activations during CA^16^ and other parasomnia events^38^, suggests possible involvement of limbic and memory circuits^40^. The combination of limbic activation with prefrontal deactivation during parasomnias may impair rational evaluation of emotional reactions, affecting the social appropriateness of behaviors (such as an inability to process others’ emotions) and increasing the potential risks associated with parasomnia enactment.

### Differences and similarities between wakefulness, sleep, and parasomnias in the theta band

The similarities between theta and delta localized activation during parasomnias, compared to sleep, are intriguing. The lower centro-parietal theta power during PE compared to sleep suggests the potential for a signature of parasomnia behaviors. Given that our sample was balanced in terms of parasomnias originating from both N2 and N3 sleep stages, we can conclude that this difference is not driven by interactions between stage and frequency band. However, the cluster showing decreased theta in PE compared to sleep is more centro-occipitally distributed compared to the cluster differentiating these states in the delta range. Additionally, theta power was significantly higher during parasomnias compared to wakefulness across the entire scalp, indicating that theta activity exhibits reduced regional specificity relative to delta when differentiating cortical sleep and wake states. Further investigations could clarify the role of delta versus theta oscillations in the clinical features of parasomnia manifestations.

### Differences and similarities between wakefulness, sleep, and parasomnias in high frequencies

The absence of significant differences in alpha activity between wakefulness and parasomnias provides further evidence supporting the wake-like nature of parasomnia episodes. Moreover, the similarities observed in the beta band between parasomnias and wakefulness, particularly over centro-parietal regions, reinforce the presence of a dissociative state. These findings align with increased beta connectivity for symmetric inter-hemispheric networks (including frontotemporal, parietal, and occipital areas) observed 20 s before episode onset in other studies^39^. Similarly, elevated beta activity in motor and mid-cingulate cortices has been observed 4–5 s before sleepwalking episodes^15^.

Interestingly, higher beta activity was found when comparing parasomnias with sleep 8 s before awakening at another point in the night, yet matched for consciousness features, as well as between parasomnias and sleep immediately preceding the parasomnia event by 10 s. However, despite using rigorous data cleaning techniques, beta findings may still be influenced by movement artifacts (including ocular movements) in our recordings. This limitation could explain the marginal increases in beta activity observed in the frontal and occipital regions during parasomnias compared to wakefulness.

This study is subject to a limitation related to parasomnia episodes recorded during daytime recovery sleep following sleep deprivation. Both sleep deprivation and circadian timing may influence spectral characteristics of the signal and may therefore have contributed to the observed spectral patterns. The use of recovery sleep was dictated by feasibility considerations, as sleep deprivation currently represents the most effective experimental approach to increase the likelihood of capturing parasomnia episodes under controlled conditions. Importantly, the overall pattern of results is consistent with previous studies based exclusively on baseline nocturnal sleep.

Whereas the acquisition of large baseline-only datasets remains challenging due to the low probability of recording spontaneous parasomnia episodes under these conditions, future studies focusing on parasomnia events occurring solely during baseline nocturnal sleep, without prior sleep deprivation, would be desirable.

## Conclusions

Historically, parasomnias were often regarded as “diabolic manifestations,” “diseases of volition,” or as expressions of the body controlling the will. However, scientific advancements have reshaped these perceptions, now classifying parasomnias as disorders of arousal. These disorders are believed to be underpinned by genetic and specific epigenetic etiologies, although the exact mechanisms remain largely unknown. The diagnostic approach has become clearer over time, with potential treatments including pharmacological and psychobehavioral therapies, highlighting the evolutionary significance of these conditions.

The primary goal of this study was to characterize the cognitive and behavioral manifestations typical of various parasomnia types and to identify their neural correlates. Our findings support the dual nature of parasomnias, showing that these episodes involve both wake-like and sleep-like states. Additionally, the data suggests that a reduction in slow-wave activity (SWA) could serve as a potential biomarker for identifying individuals at higher risk of parasomnia episodes during sleep. Furthermore, our study underscores a dissociation between heightened motor and limbic activation, coupled with relative prefrontal deactivation during parasomnia behavioral episodes. This dissociation could help explain the increased risk of self-injury and harmful behaviors commonly associated with parasomnias.

Looking ahead, future research in larger and more diverse samples is necessary to enhance our understanding of parasomnias. A more comprehensive exploration of subtypes of parasomnia episodes, which may vary in phenotype and complexity, is crucial. Future studies employing causal manipulations, such as neuromodulation approaches that selectively suppress or enhance slow-wave activity, will be useful to directly test the causal role of slow waves in parasomnia pathophysiology.

Additionally, the application of updated preprocessing techniques, such as adaptive mixtures of independent component analysis (ICA) and machine learning approaches, could further refine the insights derived from complex datasets. Such studies could deepen our understanding of the neural mechanisms underlying parasomnias and expand the translational potential of our findings, ultimately leading to more effective diagnostic and therapeutic strategies.

## Methods

Ethical approval for this study was granted by the University of Wisconsin–Madison Institutional Review Board. The experiment was performed in accordance with the relevant guidelines and regulations on research involving human subjects.

### Characteristics of study participants

Both control and parasomnia subjects provided informed consent for participation in the experiment. Participants were screened through an initial phone contact, during which sleep habits information was collected and exclusion criteria, such as medical conditions, substance abuse, and family history of certain disorders, were assessed. Exclusion criteria included conditions like alcohol or drug problems, diabetes, heart diseases, cancer, hearing impairments, obesity, pregnancy, or a family history of bipolar disorder. Additionally, individuals who had a history of electroconvulsive therapy (ECT) or transcranial magnetic stimulation (TMS) were excluded.

A further medical interview was conducted in the laboratory, where participants completed various questionnaires addressing sleep disorders (including parasomnia, insomnia, restless leg syndrome, and sleep apnea), as well as the Epworth Sleepiness Scale (ESS) to assess habitual sleepiness, the Fatigue Severity Scale (FSS) for fatigue, the Inventory of Depressive Symptomatology for depression, and a modified version of the Penn State Worry Questionnaire for worry. The Paris Arousal Disorder Severity Scale (PADSS) was also administered to assess the severity of parasomnia episodes, based on their complexity and frequency, together with the Wisconsin Sleep History Questionnaires’, which assess symptoms related to sleep paralysis during the transition into sleep, hallucination-like experiences occurring at sleep onset or upon awakening, unintended episodes of falling asleep in routine daily contexts (including meals, conversations, or sedentary situations), and the presence of vivid, dream-like experiences surrounding sleep onset or awakening. Health-related quality of life was obtained with the SF36 questionnaire (36-Item Short Form Health Survey). A detailed exploration of medical, surgical, and social history was also performed to identify potential risk factors that could confound the study results.

**Fig. 4:**
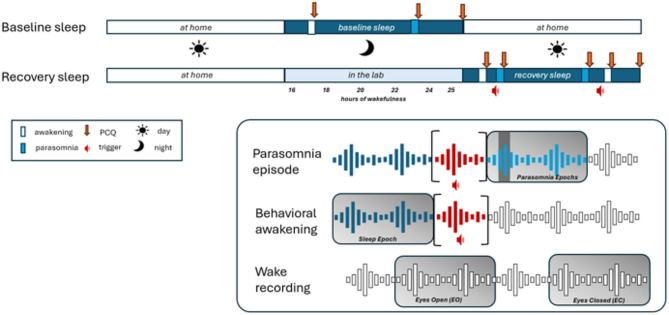
Experimental procedure and EEG comparisons. Top part. Baseline sleep involved a high-density EEG (hdEEG) and PSG (polysomnography) recording during subject habitual sleep timing in the sleep laboratory. In case of awakening or parasomnia episode, the Parasomnia Consciousness Questionnaire (PCQ) was administered to the participant, in order to assess participants’ consciousness states prior to waking up. The second night occurred within a two-week period following the baseline session, immediately after participants had been sleep-deprived for 25 h. Participants came in the lab at night, were equipped with hdEEG, had several wake EEG recordings, then went to sleep. Auditory stimulation was used to trigger parasomnia episodes, but it also triggered regular awakenings. PCQs were administered as well. Bottom part. Grey boxes indicate the EEG activities that we compared. Brain activity during parasomnia episodes ( with exclusion of the 3s auditory trigger and any section contaminated by too much movement), brain activity during sleep (that we selected just before normal—triggered or not—awakenings, in order to match the sleep stage, and experience during sleep to the parasomnia), and finally brain activity during wake (selected at 18 h of wakefulness).

Subjects who met the inclusion criteria were enrolled and scheduled for two sleep high-density EEG (hdEEG) recordings: one baseline sleep night and one recovery night following 25 h of sleep deprivation (as shown in Fig. 4). Prior to each recording, participants kept a sleep diary and were instructed to refrain from extensive travel (across more than three time zones) in the month leading up to the study and to avoid caffeine or napping on the recording day. The frequency of parasomnia episodes was not specified due to potential inaccuracies in reporting.

The participants were categorized according to the International Classification of Sleep Disorders (ICSD) criteria: current parasomnia disorder, DoA [exhibiting sleepwalking episodes, confusional arousals, (CA), sleep talking, sexsomnia on a regular basis)], past DoA (with a higher prevalence during childhood and occasional episodes in adulthood), or sleep-talking (ST) participants only. In total, 22 parasomnia participants completed the study.

### Sleep recordings

In addition to the hdEEG, recorded through a 256-channel system from Electrical Geodesics Inc., Eugene, OR, or a Neuvo amplifier from Compumedics, further monitoring was performed using EOG, submental EMG, and ECG. Electrode impedance values were recorded both before and after the sleep session.

For both recording sessions, sleep staging was performed both online and offline by a registered AASM-certified polysomnographic technician in 30-s epochs, following standard criteria^30^. Sleep staging was conducted using the Profusion Sleep EEG Software V4 (Compumedics), based on EOG, submental EMG, and six hdEEG channels placed at approximately 10–20 locations (F3, F4, C3, C4, O1, O2), with re-referencing to the mastoids. All scoring was reviewed by a board-certified sleep physician. Typical sleep variables, such as sleep efficiency and heart rate, were derived from the scoring and night review, as indicated in Table 2.

#### Baseline sleep

The baseline night involved standard monitoring with tools such as electrooculogram (EOG), submental electromyogram (EMG), electrocardiogram (ECG), bilateral tibial EMG, respiratory inductance plethysmography, pulse oximetry, and a position sensor. However, six subjects followed a slightly modified protocol where full polysomnography (PSG) was not required at the baseline assessment. Recording sessions were timed to align with the participants’ regular sleep patterns, and lights were turned off within an hour of their usual bedtime. Subjects had the freedom to sleep for as long as they wished but were required to complete the Parasomnia Consciousness Questionnaire (PCQ) whenever they woke up spontaneously during the night or after the lights were turned on in the morning. Initially, administering PCQs during baseline sleep was not permitted; however, this restriction was later adjusted to allow for PCQ administration in cases of spontaneous parasomnia events during the baseline session.

#### Recovery sleep

The recovery sleep recording occurred within a 2-week period following the baseline session, immediately after participants had been sleep-deprived for 25 h. Participants woke up at home on the day of this second part of the experiment, experienced a regular day at home or at work, and then came to the sleep laboratory nine hours before their calculated bedtime for the recovery sleep. The final stretch of this sleep deprivation period was thus closely monitored in the sleep laboratory and included four cognitive testing sessions, conducted every 2 h, starting after 16 h of wakefulness.

This protocol aimed to induce parasomnia episodes by increasing sleep pressure (due to sleep deprivation) in an unfamiliar environment. Most of the participants (*n* = 11) reported an increased likelihood of parasomnia episodes when sleeping in a new environment, or when extremely fatigued (*n* = 11). When participants reached stable N3 sleep, they were awakened by a three-second, 1000 Hz tone at 40 dB. If the initial attempt was unsuccessful, a 2-min wait occurred, followed by an increase in volume by 10 dB in the next attempt, repeated up to a maximum of 90 dB.

In the rare case that a subject did not wake up after the 90 dB tone, the researcher would restart the process at 40 dB, calling the participant’s first name while the tone was played through the headphones. If a behavioral response occurred, the PCQ was administered following the awakening or parasomnia event, and participants were allowed to return to sleep. This sleep deprivation and forced-arousal paradigm, which deepens sleep while introducing external disruptions, is an established procedure designed to induce parasomnia episodes, following a protocol established by Pilon et al. 2008^28,29^.

Participants were allowed to sleep as long as they wished, with a minimum of six hours in bed encouraged. The PCQ was administered one final time when the lights were turned on at the end of the recording session.

### Parasomnia consciousness questionnaire

The Parasomnia Consciousness Questionnaire (PCQ) is a tool designed to assess participants’ consciousness states prior to waking up, either from normal sleep or during parasomnia events. In line with Siclari et al.^27^, consciousness was defined when participants reported having a subjective experience immediately before awakening.

The PCQ gathers detailed information regarding the nature of the experiences, the participant’s level of sleepiness upon awakening (assessed using the Karolinska Sleepiness Scale, KSS), and whether those experiences were dream-like or thought-like. Participants also evaluate the level of sensory perception involved and note if they recognized specific elements such as faces, speech, self-representation or felt a sense of control, as well as any movements experienced. Additional questions probe awareness of their recent behaviors and whether the experiences reminded them of specific places or events.

These questions were administered whenever participants wake up spontaneously during either the baseline or recovery sleep recordings, as well as after any parasomnia events triggered by the experimental procedure. If participants did not wake up spontaneously following a parasomnia event, they were awakened to provide their responses to the PCQ. To ensure comfort with the questionnaire, participants were given a copy during their initial medical interview and familiarized with the questions before their subsequent visits to the sleep lab.

### EEG preprocessing

**Fig. 5:**
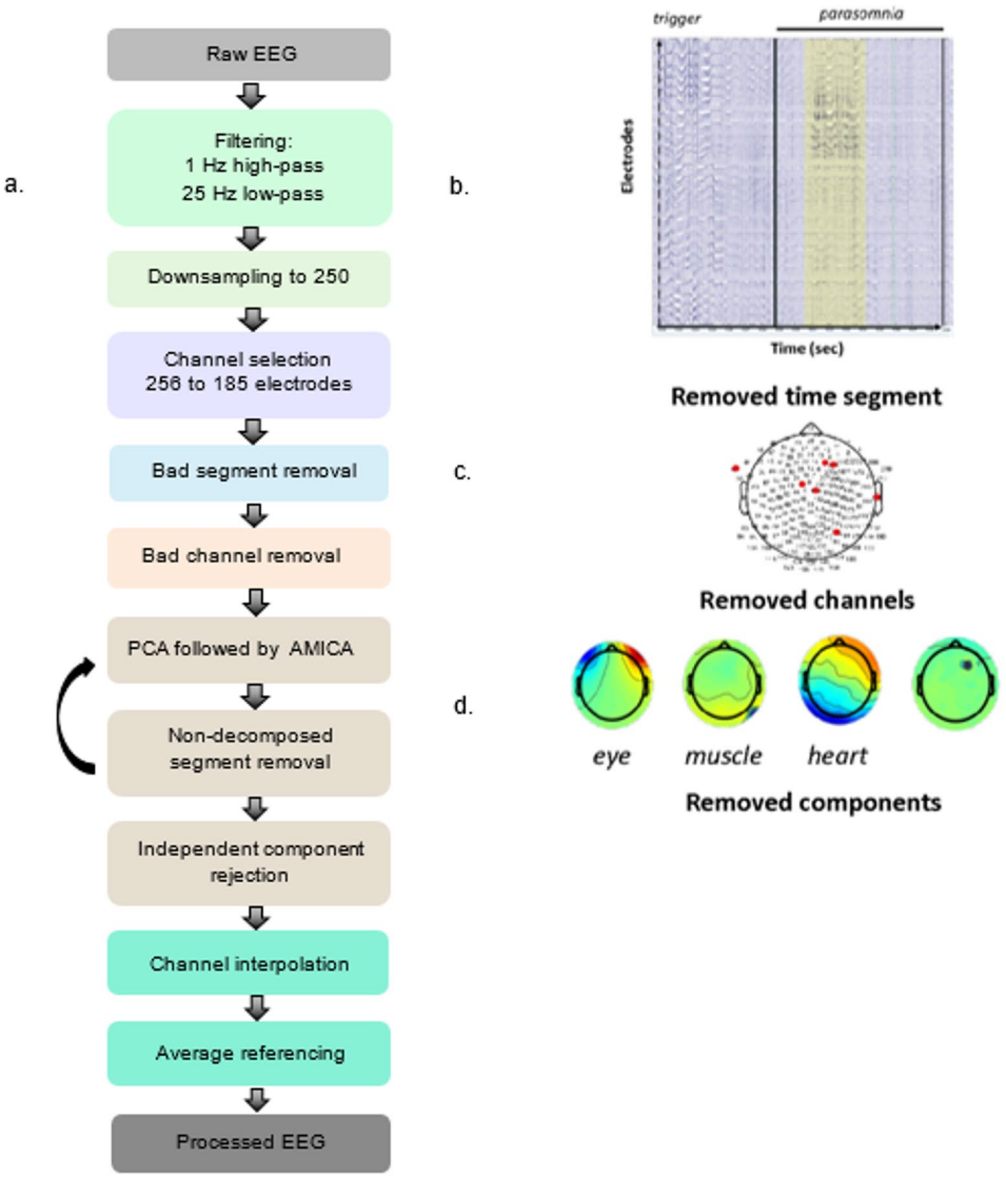
Parasomnia preprocessing pipeline. (**a**) The raw data of all 256 electrodes was filtered and then down-sized to 185 electrodes. A semi-automatic step of bad segment and bad channel removal followed. PCA (Principal Component Analysis) followed by AMICA (Adaptive Mixture Independent Component Analysis) was performed to remove clear artefactual components (from eyes, muscles, heartbeats or channels). Data for missing electrodes was interpolated from neighboring channels, and signal was re-referenced to an average reference. (**b**) Short extremely noisy time-segment were removed for subsequent data analysis. (**c**) Topographical representation of extremely artifactual channels removed from recordings for subsequent analysis. (d) Examples of removed ocular, muscular and eletrocardiographic noise components identified applying ICA (Independent Component Analysis) to the signal.

Figure 5 describes the preprocessing pipeline. To improve the signal-to-noise ratio, all EEG recordings were reduced from the full 256 channels to an inner set of 185 electrodes, excluding those placed on the face and neck. The data was collected at 500 Hz and high-pass filtered at 0.1 Hz. Full-night EEG data was offline bandpass-filtered between 1 and 30 Hz and then downsampled to 250 Hz. Channels with artifacts affecting the majority of the recording were visually identified and removed. Additional spectral-based and topographic methods were employed to remove channels with anomalous power compared to neighboring channels. Overall, less than 25% of the channels were removed and subsequently interpolated using spherical interpolation. Frequencies above 25 Hz were excluded to avoid muscle and powerline artifacts. Data preprocessing was carried out using the EEGLab toolbox in Matlab, along with in-house Matlab scripts and additional graphic interfaces, such as the ‘csc_EEG_plotter’ (developed by Armand Mensen for sleep data analysis).

#### Parasomnia epochs

Following preprocessing, full-night recordings were carefully segmented to capture neural dynamics surrounding the 79 recorded parasomnia episodes, preserving a 15-min window before and after each event. The onset of each episode was determined by identifying the first discernible deviation in the EEG signal, the submental EMG trace, or overt behavioral markers such as movement or eye opening observed on video recordings. Each extracted epoch was meticulously reviewed, with stretches of excessive noise or compromised signal quality discarded to ensure the integrity of the analysis.

To further refine the data, Independent Component Analysis (ICA) was applied using the ‘Runamica’ algorithm implemented in the EEGLAB toolbox. This step systematically removed physiological and external electrical noise, including ocular and muscular artifacts, electrocardiographic contamination, and channel-specific distortions for each event and patient separately. Once ICA was performed, time courses of resulting independent components were visually inspected, and data segments in which noise pervaded multiple components were excised. A second ICA iteration was subsequently conducted, targeting residual sources of physiological or artifactual interference—such as head movements or line noise—before the data was re-referenced to the average of all channels.

Parasomnia episodes were classified according to their sleep-state of origin. Of the 79 documented events, six emerged from REM sleep across two subjects, five of which were characterized by sleep talking, while one was identified as a complex confusional arousal. The remaining 73 episodes arose from NREM sleep and exhibited distinct phenomenological features. Sleep-talking episodes were marked by minimal bodily movement and extended speech, often with the eyes closed. Confusional arousals, by contrast, ranged in complexity from brief utterances and isolated movements to elaborate, coordinated behaviors in bed, sometimes accompanied by speech. Although nine NREM episodes were excluded due to excessive movement artifacts, data from the pre-episode period was successfully preserved for all 79 cases. A detailed characterization of these episodes is provided in Tables 3 and 4, which includes a description of the features of the parasomnia episodes.

**Table 4:** Details on PE behavioral manifestations and dream reports. The table presents the behavioral response and the dream report (when present) of 79 PE (parasomnia episodes) within the experimental setting. 36 PE presented with consciousness upon awakening are described and categorized. The third column presents specific features of the PE such as eyes opening, head or body movements, purposeful arm movements, simple vocalizations or complex sleep talking events. The last column presents the description of the dream related to the PE episode provided by patients immediately upon awakening, administering PCQ to gather detailed information about the experience report (CE, conscious- experience in Table 3).

Subject	Unique parasomnia code	Description of PE behavioural response	Description of PE dream report
P001	60	She opens eyes and briefly looks around, after a jerky body movement	
P002	86	He smiles and laughs softly	
	88	He lifts his head up	
	90	He reaches for the headnet adaptor	
P003	128	She mumbles unintelligibly	
	135	She lifts her head up and says ‘yeah’	
P004	34	She opens eyes, says “Hmm?” while stretching her arm and extending her fingers	
	32	She opens eyes, lifts her hands close to face and moves her fingers around, while saying “Hmm, I can’t”	
	33	She opens her eyes, lifts her hands close to face and moves her fingers around, then pronounces “but nothing that wouldn’t let me…”	
	28	She opens eyes, turns head and says “Hmm?”	“I was thinking of zebras”.
P005	46	He turns in his bed and rearranges pillows	
	51	He sits up on elbow while saying “Wow” and taping his leg	“It was something about money. Somebody wanted money from me. But I didn’t have it right away. That’s why I said… I remember what I said, I said - Wow”
	43	He screams “f*** this ***” while throwing his pillow onto the ground, then grabs his headnet	“I felt like a buzz sensation. In response to question about emotions maybe, he says: I don’t even remember to be honest.”
	52	He looks around and taps his pillow three times	“I was going to golf with someone, I was waiting for them. In my mind, or I don’t know if it was in the environment, I thought I could see shadow of someone getting ready to golf with a golf bag (on the backwall of the room)”.
	41	He yells “ah f***off"and “get the f*** out here”, throws pillow and then curls in bed, recovering	“I think someone was sitting in the rocking chair. I think that’s what happens. I think it was a kid wearing red. He didn’t do anything, just freaked me out.”
	45	He says, “I need to be sitting up right? Give a second…or maybe four seconds”	He then remembers that it was about me coming in the room to fix his electrodes.
	49	He pushes something away, smiles, gives a thumbs up and then laughs	Initially says there was no consciousness, but after describing, he remembers more, but his report is confused. “I remember … unclear … I’m sorry, I’m out of it right now.”
P006	77	He props himself upright and adjusts net cable	“I was teaching to a classroom full of kids, maybe 30-40. There were two groups of kids, but I was only worried about mine. Then it was more outdoors, I was trying to keep them all bundled together, like at daycare when they go out and have to keep an hand on a string to prevent them from wandering off. Then at the end of the dream, they all broke free, running in every direction.”
	68	He lifts his head up	
	74	He looks around and props up on right elbow	“Just before that, there was… »15s later: felt there was something but II really just don’t know”.
	69	He lifts his head while softly saying “Hmm”	
	75	He looks around and says “is this audible to you, this sound just before the beep, which souds like lala la la lala”	“Feels like I had woken up briefly, and tried to communicate it to you that I had woken up, and nothing worth, trying to go back to sleep, but instead of communicating that in a meaningful way, I just kind of, I don’t know, said some garbage and then went back to sleep.”
	78	He waves his hand and says “check out the big bus - bouth -, we went cause of the last big fight”	“Dreaming for a while, in and out, just… all taking place in warmer climate. Like South america or Wisconsin in legitimate summer, came back to idea of keeping children together with rope, getting them going in one direction, cause if you lose one, you have to get him back, etc… lots of people involved, lots of adults.”
P007	114	He says “hm-um”	“I was actually dreaming about the last time you woke me up and answering questions. I thought I was still thinking and awake since the last time you asked me questions.”
	111	He lifts his head up, turns it and says, “hmm”	
	113	He says, “hmm” “what do you mean, how short?”	“I was thinking I was talking to someone. I was being told to answer questions, specifically about how I was feeling.”
P008	160	He says “I was what?”	“Yeah, I thought that you were still asking me questions and waiting for a response and I had forgotten what I said.”
	156	He says, “yes, hmm… Hello?”	
	159	He looks up and says, “I didn’t know what it was…. It was mmmmeh”	
P009	10	He says “yeah”	“I think I was having a picnic and cooking sausages with people.”
	5	He says “Hmm” and wakes up	
	4	He sighs and says “yeah?”	“Maybe thinking of buckets of water standing upright or something.”
	8	He says “well,…, I don’t know if I was sleeping, but I’m not sure”	
P010	124	She lifts her head up and looks left to right	
	126	She moves her blanket and touches the headphones	
P011	11	He lifts his head up and looks around while sighing	
	12	He looks left and right and says, “Hmm what’s that?“, and says “Oh man”	
	17	He looks around and touches the headnet and says ‘hmm?’	
	19	He lifts his head up, looks around and says “Ok”	
	15	He looks around and says, “hmm, yup ok I’m going there”	
	18	He sighs and reaches for phone	
P012	143	She props on elbow, looks around and repeats a sentence twice (“las latitas, las latitas” not intelligible)	
	140	She mumbles unintelligibly	
	148	She opens her eyes and says “Sí?”	
	147	She opens her eyes	
P013	182	She looks around and says “Hello, yep yeah…”	“No. Well, actually hmm, the greenery one. We’re like a few… [mumbles some words]. Actually I’m completely confusing and getting it confused. In my textiles class, it’s the piling test for carpet, that’s what I was thinking of.”
	184	She says, “… a high pitched sound…straight”	She remembers waking up to a sound, vaguely remembers talking about the sounds that we play in her ears. She says she’s sure she talked about the noise “we guys” are playing in her ears
	178	She says, “I’m moving toward you if that makes any sense.”	
	180	She moves around in bed and says “yeah.really heavy”	
	176	She touches her face and whispers	
	177	She stretches, checks phone, and asks, “Is everyone okay?”	“I was dreaming, I don’t know, about something falling or whatever, was about to fall on someone. People were gasping in my dream and I asked if everyone was ok.” (Later at question on emotion, she remembers about a brick wall about to fall on people, and she remember saying “Is everyone OK?“)
P014	186	He turns his head side to the side and mumbles “oh” while laughing	“I was having a weird dream, a car driving, either watching or in the car, it was moving, but unsure if anyone in it. On a road or highway.”
	190	He says “three”	“I was thinking of the tail of one of the PCQ question, - OK, now, you’re gonna go back to sleep,- voice through the headphone.”
	189	He looks around and says, “I forgot to do something but I can’t remember what it was.Ah yeah”	Initial response is no recall. Then he said he was supposed to be doing something and fell asleep
P015	252	He looks around	“A lot of books”
	254	He says “Hmm-hm” like acquiescing and scratch his neck	
	255	He says “Hmm…” then “Yeah” then he sighs and stretches his body	Explaining the rules of the office
	257	He says “hm-hmm with small body movements (like acquiescing)	
	256	He says “Hmm? Okay. One…” then “zero”	“Answering a question about the scales and realized not actually……”
	271	He says “No”	
	274	He says “Hmm?”	
P016	291	Whispers “stop, stop” and “oh jeez!”	
	292	She stretches her right arm out and whispers	
P017	338	She looks around.	
P018	348	He says “wow”	
	351	He moves his head and mumbles unintelligibly while appearing to clean off something from the bedsheet	“Thought there was a spider on the bed and crumbs (like bread crumbs or baking crumbs).”
	354	He looks around while lifting pillow with one hand	
	356	He moves his arm under the blanket in the same manner as in 351	
	359	He picked at his shirt with hand while mumbling and putting the pillow on his head	Dreaming about football, he was in Omaha
	360	He says “three out of four”	
	352	He says no	
P019	374	He says “yes” and taps his fingers three times on the bed	He was thinking that we were asking about his dreams and events and was answering our questions
	375	He says “three”	He was answering another one of our sliding scale questions with “3”
	378	He says “no”	Dreaming about answering questions from the last conversation that they had
	366	He says “three”	Thinking about movement
	367	He looks around and utters “Hmm”?	Thinking about something and came to some realization which made him say “oh!” which woke him up
	368	He says “no”	Thought he was answering questions
P020	385	She says “zero”	Dreaming about telling us about a parasomnia event, said “zero” to answer one of our questions
	379	She half sits up and says “uhm”	Having a dream about talking to us over the intercom

#### Sleep epochs

In order to compare brain activity during parasomnia to brain activity during sleep, full-night recordings were trimmed to isolate relevant data segments (as shown in Fig. 4). We aimed at taking sleep epochs which preceded normal awakenings to which PCQ was administered. Out of 315 recorded awakenings, 46 were successfully processed and paired with 62 parasomnia episodes, as detailed in Supplementary Table S1. In eight cases, no suitable match was available within the same subject and night (Table 3, Supplementary Table S1). Notably, 15 awakenings served as reference points for more than one parasomnia episode.

Matching criteria prioritized awakenings that shared the same sleep stage and reported level of consciousness—conscious or unconscious—as the parasomnia event to which they were paired. Each matched awakening was drawn from the same night and was selected to be as temporally proximal as possible to the corresponding parasomnia event. For NREM sleep analyses, epochs corresponding to N2 and N3 sleep were selected, with sleep periods defined according to the framework proposed by Feinberg and Floyd 1979^31^. Given the disruptive nature of the study protocol, particular attention was paid to the delineation of sleep cycles. Preprocessing steps—including artifact rejection and ICA cleaning—were implemented identically to those applied to parasomnia episodes, preserving methodological consistency.

#### Wake epochs

To establish a baseline against which sleep and parasomnia events could be compared, standardized wake EEG recordings were obtained across multiple time points during the sleep-deprived night (as shown in Fig. 4). The protocol included an initial session at the start of the night, another one after 16 h of sustained wakefulness, followed by recordings at 18, 20, and 22 h awake, with a final session conducted immediately before recovery sleep.

During each recording, subjects remained seated in a controlled environment, alternating between 2-min epochs of eyes-open (EO) and eyes-closed (EC) conditions. In the EO condition, subjects fixated on a cross displayed on a monitor, while in the EC condition, they rested with their eyes closed. The resulting EO and EC segments from each of the recording session were concatenated, after which artifact-contaminated channels and data stretches were systematically removed. ICA processing was performed separately for each condition (EO/EC) and for each subject. Spectral analyses were subsequently conducted on the initial wake EEG recordings to provide a reference for comparisons with sleep and parasomnia events, while also confirming that wakefulness data remained free of excessive sleep pressure or contamination.

### Statistical analyses

Spectral Power Density (PSD) was computed using Welch’s modified periodogram method, implemented via the ‘pwelch’ function in Matlab (MathWorks Inc., Natick, MA). The analysis employed 2-second Hamming windows with 50% overlap, facilitating the decomposition of neural signals into distinct frequency bands: delta (1–4 Hz), theta (4–8 Hz), alpha (8–12 Hz), sigma (12–15 Hz), and beta (15–25 Hz).

All statistical analyses were performed in Matlab. Within-subject comparisons were conducted using two-sided paired t-tests to examine differences between behavioral states. At the group level, average spectral power values were analyzed indipendently for each frequency band. To correct for multiple comparisons across the scalp, we employed a nonparametric, cluster-based permutation approach, utilizing Threshold-Free Cluster Enhancement (TFCE) and Statistical Nonparametric Mapping (SNPM)^32^. Brain activity during PE was compared to both sleep recordings preceding normal awakenings and wakefulness recordings. Specifically, absolute power values were averaged across 185 derivations for each condition—sleep, parasomnia, and wake—within each frequency band. Paired two-sample, nonparametric, one-tailed t-tests were then applied independently for each frequency band, contrasting parasomnia with wakefulness and parasomnia with sleep, while preserving the “consciousness” matching outlined in the “Methods” section. Wake recordings from each participant were individually paired with their corresponding parasomnia events.

To further validate statistical robustness, TFCE was applied with weighting parameters E = 2/3 and H = 2. Power values were thresholded at a corrected significance level of *p* < 0.05^33^.

## Supplementary Information

Below is the link to the electronic supplementary material.

Supplementary Material 1

## Data Availability

The datasets generated during and/or analyzed during the current study are available from the corresponding author upon reasonable request.
