# Supplementary material for: High-density EEG signature of NREM sleep parasomnia episodes
Source: Sci Rep. 2026 Apr 27;16:19414. doi: 10.1038/s41598-026-41601-4 (PMC13287612; doi:10.1038/s41598-026-41601-4)
Supplement: Supplementary file 1 — Supplementary Material 1 [file 41598_2026_41601_MOESM1_ESM.pdf]

High-density EEG signature of NREM sleep parasomnia episodes

A. Valomon[1] – I. De Cuntis[1]**, K. Nakamura[1], B. A. Riedner[1,2], S. Jones[1,2], M. Bazalakova[1,2,3], D. T. Plante[1,2], G. Tononi[1] and M. Boly*[1,3]**

[1]Psychiatry - Wisconsin Institute for Sleep and Consciousness, University of Wisconsin-Madison, Madison, WI, 53719, USA
[2]School of Medicine and Public Health, University of Wisconsin-Madison, Madison, WI, USA
[3]Neurology, University of Wisconsin-Madison, Madison, WI, USA

*Corresponding author: boly@neurology.wisc.edu
** the authors contributed equally to this work.

Supplementary Material

Suppl. Fig S1 Subdivision of parasomnia subjects based on their behaviors

A gradient on some sleep variables tended to occur between the different sub-groups: current DoA subjects (13), past DoA subjects (6), sleeptalkers only (3), and controls (12). Tests were performed between NREM parasomnia group (n=22) and controls (n=12) only: * indicate significant differences. Details of the questionnaires codes are reported in Table 1.

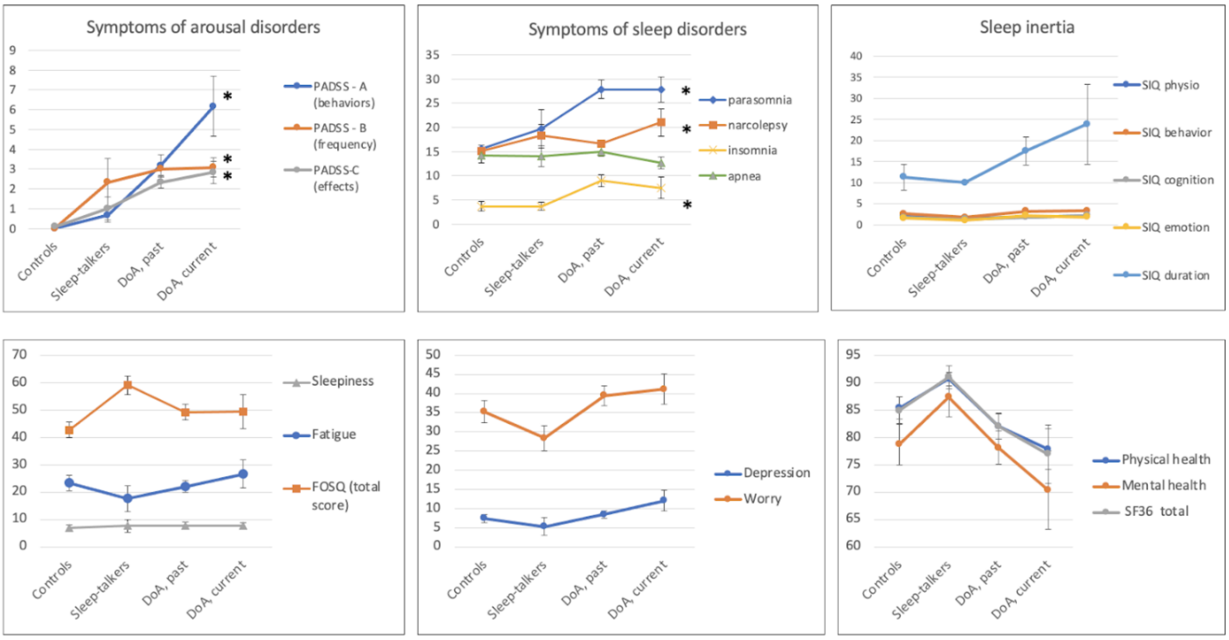

Suppl. Table S1 Parasomnia episodes with paired sleep

Matching of parasomnia episodes with awakenings from sleep from the same subject, as much as possible from the same night origin, sleep stage and subsequent consciousness report. (CE for consciousness, N for No consciousness, CNR for Consciousness with no report, . for missing report). Awakenings were as close in time as possible to the PE. Strikethrough unique PE codes indicate unusable parasomnia.

Subject	Night	Sleep stage	Unique Parasomnia code	Consciousness Report	Unique Awakening code	Night	Sleep Stage	Consciousness Report
P001	RS	N2	~~60~~	N	59	RS	N2	N
P002	RS	N3	86	.	84	RS	N3	N
	RS	N3	88	N	85	RS	N3	N
	RS	N3	90	N	87	RS	N3	N
P003	BS	N3	128	.	129	BS	N2	N
	RS	N3	135	N	136	RS	N3	N
P004	RS	N3	34	N	27	RS	N3	N
	RS	N2	32	.	29	RS	N2	N
	RS	N3	33	N	30	RS	N2	CE
	RS	N2	~~28~~	CE	35	RS	N2	CE
P005	RS	N3	46	N	48	RS	N3	N
	RS	N2	51	CE	53	RS	N2	N
	RS	N3	43	CNR	48	RS	N3	N
	RS	N2	52	CE	53	RS	N2	N
	RS	N3	41	CE	48	RS	N3	N
	RS	N2	45	CE	53	RS	N2	N
	RS	N3	~~49~~	CE	48	RS	N3	N
P006	RS	N3	77	CE	73	RS	N3	CE
	BS	N3	68	.	70	BS	N3	CE
	RS	N3	74	CNR	73	RS	N3	CE
	BS	N3	69	.	70	BS	N3	CE
	RS	N3	75	CE	76	RS	N2	CE
	RS	N3	78	CE	79	RS	N2	CE
P007	RS	N2	114	CE	112	RS	N2	CE
	RS	N2	111	.	112	RS	N2	CE
	RS	N3	113	CE	116	RS	N2	CE
P008	RS	N2	160	CE	157	RS	N3	CE
	RS	N3	156	N	168	RS	N3	N
	RS	N2	~~159~~	N	173	RS	N2	N
P009	RS	N3	10	CE	9	RS	N2	CE
	RS	N2	5	N	9	RS	N2	CE
	RS	N3	4	CNR	6	RS	N3	CNR
	RS	N3	8	CNR	7	RS	N3	CNR
P010	RS	N3	124	N	123	RS	N3	N
	RS	N3	126	N	125	RS	N3	N
P011	BS	N3	11	.	13	BS	N2	N
	BS	N3	12	.	13	BS	N2	N
	RS	N3	17	N	16	RS	N2	N
	RS	N3	19	N	20	RS	N3	N
	RS	N3	~~15~~	N	20	RS	N3	N
	RS	N3	~~18~~	N	24	RS	N3	N
P012	RS	N3	143	N	144	RS	N3	N
	RS	N3	140	N	144	RS	N3	N
	RS	N3	148	N	149	RS	N3	N
	RS	N2	147	N	151	RS	N2	N
P013	RS	N3	182	CE	179	RS	N3	N
	RS	N2	184	CE	181	RS	N3	N
	RS	N3	178	N	179	RS	N3	N
	RS	N3	180	N	181	RS	N3	N
	BS	N3	176	.				
	BS	N2	177	CE				

P014	BS	N3	186	CE	187	BS	N2	CNR
	RS	N2	190	CE	193	RS	N2	CE
	RS	N3	189	CE	197	RS	N3	CE
P015	BS	N3	252	CE	251	BS	N3	CE
	BS	N2	254	N	263	BS	N3	N
	BS	N3	255	CE	251	BS	N3	CE
	BS	N2	257	N	263	BS	N3	N
	BS	N2	256	CE	251	BS	N3	CE
	RS	N3	271	N	268	RS	N3	N
	RS	N3	274	N	272	RS	N3	N
P016	BS	N2	291	N				
	BS	N3	292	N				
P017	BS	N3	~~338~~	N	339	BS	N2	N
P018	BS	N2	348	N	349	BS	N3	N
	BS	N2	351	CE	349	BS	N3	N
	RS	N3	354	N	357	RS	N2	N
	RS	N3	356	N	361	RS	N3	N
	RS	N3	359	CE	363	RS	N3	CE
	RS	N3	360	N	364	RS	N3	N
	BS	N2	~~352~~	N	349	BS	N3	N
P019	RS	N3	374	CE	372	RS	N3	CE
	RS	N2	375	CE	376	RS	N3	CE
	RS	N2	~~378~~	CE	376	RS	N3	CE
	BS	N2	366	CE				
	BS	N2	367	CE				
	BS	N2	368	CE				
P020	RS	N2	385	CE	387	RS	N2	CE
	BS	N3	379	CE				

Supp. Table S2

Details on parasomnia episodes with conscious experience

36 PE presented with consciousness upon awakening (divided into CE and CENR). The table presents each parasomnia (subject, origin (baseline or recovery sleep), type of parasomnia (ST, CA), a rating on the amount of sounds (ST) and movement (MVT) (see legend to table 3). Sleepiness upon awakening was obtained from the KSS scale. Details on the parasomnia obtained via the PCQ are indicated (*reminiscence*: did the episode remind the participant of anything already experienced?, *awareness*: were they aware they just had an episode?, *emotions*: were they having negative (-2) or positive emotions (+2), other scales from 0 to 4 (strongest): *dreamlike experience:* were they aware they were dreaming, *control*: were they in control of what they were doing, *perception*: how much were they perceiving things (seeing/hearing), *thinking*: how much were they thinking during their experience, *faces*: did their experience involve faces, *speech*: did their experience involve speech, *yourself*: how much was their experience centered on themselves, *environment*: how much was it centered on their environment, *space*: were they moving through spaces, *moving*: how much were they or other people moving during their experience, *where*: where did your experience take place). The complexity of the reported experiences in the 36 episodes with consciousness was rated from 1 to 3 based on the number of words. Category 1 indicated that participants were aware of experiencing something but could not remember the content (CNR category: consciousness with no recall). Category 2 indicated an experience that could be briefly summarized, such as thinking of zebras or talking to a sleep researcher through the intercom. Category 3 described more dream-like experiences with scenarios, including details on the environment, people, actions, etc.. Additional details on the participants' experiences included the location (indoors, outdoors, others), the type of actions (more speech, more action, thinking, observing), and the emotions felt during the experience.

PE ID	Participant	Night	Parasomnia Type	ST	MVT	Sleep stage	KSS	Consciousness	Reminiscence	Awareness of episode	12. emotions	13. dreamlike	14. control	15. perceptiono	16. thinking	17. faces	18. speech	19. yourself	20. environment	21. space	22. moving	23 where	Number of words	Complexity scale	Environment relate
4	P009	RS	CA (simple)	1	1	N3	6	CNR	na	.	na	na	na	na	na	na	na	na	na	na	na	na	0	1	
8	P009	RS	Sleep-talk	2	0	N3	5	CNR	na	**yes**	na	na	na	na	na	na	na	na	na	na	na	na	0	1	
10	P009	RS	CA (simple)	1	1	N3	.	CE	**yes**	.	1	3	3	1	2	3	3	3	1	0	1	*outdoors*	10	2	
28	P004	RS	CA (simple)	1	2	N2	9	CE	no	.	0	1	0	2	0	0	0	1	2	0	0	*outdoors*	1	2	
41	P005	RS	CA (complex)	2	3	N3	9	CE	**yes**	**yes**	-2	1	0	3	1	0	0	.	.	.	.	.	28	3	
43	P005	RS	CA (complex)	2	3	N3	.	CNR	na	**yes**	na	na	na	na	na	na	na	na	na	na	na	na	0	1	
45	P005	RS	Sleep-talk	2	0	N2	.	CE	.	no	.	.	.	.	.	.	.	.	.	.	.	.	10	2	
49	P005	RS	CA (complex)	1	3	N3	4	CE	**yes**	no	1	.	.	.	.	0	2	.	.	.	1	indoors	10	2	
51	P005	RS	CA (complex)	1	3	N2	6	CE	no	**yes**	0	1	2	1	1	0	3	3	2	1	1	indoors	24	3	
52	P005	RS	CA (complex)	0	3	N3	6	CE	**yes**	**yes**	0	2	1	3	1	0	1	3	2	0	2	indoors	40	3	
74	P006	RS	CA (simple)	0	2	N3	6	CNR	na	.	na	na	na	na	na	na	na	na	na	na	na	na	0	1	
75	P006	RS	CA (complex)	2	3	N3	8	CE	.	**yes**	.	.	.	.	.	.	.	.	.	.	.	.	52	3	
77	P006	RS	CA (complex)	0	3	N3	.	CE	**yes**	no	1	4	3	2	2	4	2	1	1	3	4	**both**	74	3	
78	P006	RS	CA (complex)	2	3	N3	6	CE	**yes**	no	1	2	3	2	1	4	2	3	0	3	4	*outdoors*	58	3	
113	P007	RS	Sleep-talk	2	0	N3	7	CE	**yes**	no	0	3	2	4	3	0	4	4	0	0	0	***neither***	18	2	yes
114	P007	RS	Sleep-talk	1	0	N2	7	CE	**yes**	no	0	3	3	4	3	0	4	4	0	0	0	indoors	12	2	yes
160	P008	RS	Sleep-talk	2	0	N2	9	CE	.	.	.	.	.	.	.	.	.	.	.	.	.	.	18	2	yes
177	P013	BS	CA (complex)	2	3	N2	7	CE	no	**yes**	-1	3	0	3	4	1	4	1	4	1	3	***neither***	29	3	
182	P013	RS	CA (complex)	2	3	N3	6	CE	**yes**	.	0	.	.	.	.	.	.	.	.	.	.	.	23	3	
184	P013	RS	Sleep-talk	2	0	N2	5	CE	**yes**	**yes**	.	.	.	.	.	.	.	.	.	.	.	.	12	2	
186	P014	BS	Sleep-talk	1	0	N3	8	CE	no	no	0	0	1	4	2	2	2	3	1	1	1	*outdoors*	27	3	
189	P014	RS	CA (complex)	2	3	N3	8	CE	.	**yes**	.	.	.	.	.	.	.	.	.	.	.	.	5	2	
190	P014	RS	Sleep-talk	1	0	N2	8	CE	**yes**	.	1	0	3	2	0	0	3	4	0	0	0	indoors	21	2	yes
252	P015	BS	CA (simple)	0	1	N3	6	CE	no	.	1	0	0	2	0	1	0	0	0	0	2	***neither***	4	2	
255	P015	BS	Sleep-talk	2	0	N3	4	CE	no	no	0	0	0	0	0	0	1	0	0	0	0	***neither***	6	2	
256	P015	BS	Sleep-talk	2	0	N2	4	CE	**yes**	**yes**	0	0	1	1	2	0	4	4	4	0	0	indoors	10	2	yes
351	P018	BS	CA (complex)	2	3	N2	7	CE	**yes**	**yes**	-2	0	3	4	3	0	0	2	4	0	2	indoors	9	2	
359	P018	RS	CA (complex)	2	3	N3	5	CE	**yes**	no	2	0	2	.	3	3	3	3	1	1	0	indoors	8	2	
366	P019	BS	Sleep-talk	1	0	N2	3	CE	no	no	1	0	4	4	3	0	3	3	3	0	3	indoors	10	2	
367	P019	BS	CA (simple)	1	1	N2	6	CE	**yes**	**yes**	0	2	3	3	4	3	4	3	2	0	1	**both**	26	3	
368	P019	BS	Sleep-talk	1	0	N2	7	CE	**yes**	.	0	2	2	4	4	0	4	4	3	0	0	indoors	3	2	yes
374	P019	RS	CA (complex)	1	3	N3	6	CE	**yes**	**yes**	0	0	4	4	4	3	4	4	0	1	3	**both**	13	2	yes
375	P019	RS	Sleep-talk	1	0	N2	5	CE	**yes**	**yes**	0	0	2	4	4	0	4	4	2	0	3	indoors	11	2	yes
378	P019	RS	Sleep-talk	1	0	N2	8	CE	**yes**	.	0	0	0	3	3	0	4	3	0	0	0	.	11	2	yes
379	P020	BS	CA (simple)	1	2	N3	8	CE	no	.	0	1	1	.	3	4	3	3	4	1	1	indoors	6	2	yes
385	P020	RS	Sleep-talk	1	0	N2	9	CE	**yes**	**yes**	0	1	3	3	4	3	4	4	4	0	0	indoors	14	2	yes
